# Relationships Between Traumatic Life Events, Cognitive Emotion Regulation Strategies, and Somatic Complaints

**DOI:** 10.1007/s10880-017-9494-y

**Published:** 2017-05-15

**Authors:** Nadia Garnefski, Yanda van Rood, Carlijn de Roos, Vivian Kraaij

**Affiliations:** 1 Department of Clinical Psychology, Leiden University, P. O. Box 9555, 2300 RB Leiden, The Netherlands; 2 Department of Psychiatry, Leiden University Medical Centre, Leiden, The Netherlands; 3 Psychotrauma Centre, MHI Rivierduinen, Leiden, The Netherlands

**Keywords:** Somatic complaints, Cognitive emotion regulation, Trauma

## Abstract

The aim of this study was to investigate the relationships between traumatic life events, specific cognitive emotion regulation strategies, and present somatic complaints. The sample consisted of 465 adults from the general population. The participants filled in online self-report questionnaires with regard to somatic complaints (SCL-90), cognitive emotion regulation strategies (CERQ) and traumatic life events. Multiple regression analysis was performed to study the relationships. The results showed that present somatic complaints were significantly related to the reporting of past negative events (such as loss and maltreatment) that still produce strong and negative feelings in the present. Somatic complaints were also significantly related to a more frequent use of maladaptive cognitive coping strategies, such as blaming oneself, ruminating, and catastrophizing about negative life events. Inquiring about unresolved traumatic memories and coping strategies can help guide a clinicians’ approach to managing patients with somatic complaints that have no clear medical explanation.

## Introduction

Somatic complaints that are commonly seen in primary medical care are abdominal pain, shortness of breath, chest pain, fatigue, and limb pain. In many cases clear medical explanations can be found and adequate medical treatment can be provided. However, research has demonstrated that for at least 33% of somatic complaints in primary care no sufficient medical explanation can been found (Kroenke, 2003; Steinbrecher, Koerber, Frieser, & Hiller, 2011). Somatic complaints highly impact someone’s quality of life. Therefore, it is important to look at other, non-medical factors, in order to find targets for assessment, referral, and intervention. One of the factors that is assumed to play an important role in the development and maintenance of somatic complaints is the experience of traumata (for reviews, see Afari et al., 2014; Brown, 2004, 2006). This has been confirmed by a number of review studies, which show that people with somatic complaints report higher traumatic event rates than controls (Roelofs & Spinhoven, 2007), that having a post-traumatic stress disorder (PTSD) is associated with a range of somatic complaints (Gupta, 2013), and that people exposed to traumatic events are 2.7 times more likely to have one or more somatic syndromes (Afari et al., 2014). In current models, such as the hierarchical cognitive model used in the present study, the ideas concerning the influence of traumata have been extended. Recently, it has been assumed that certain somatic complaints may be produced and maintained by a combination of re-activation of traumatic memories (including sensory-motor experiences) and maladaptive cognitive strategies, like rumination and catastrophizing (see Brown, 2004, 2006, 2007). Some preliminary evidence has been found by Garner (2016) who showed that rumination moderated the relationship between stressors and somatic symptoms in a sample of university students. Whether cognitive coping strategies play a role in the relationship between traumatic life events and somatic complaints, has not yet been empirically confirmed.

Cognitive coping or cognitive emotion regulation strategies can be defined as the conscious, mental strategies individuals use to handle the intake of emotionally arousing information (Thompson, 1991; Garnefski, Kraaij, & Spinhoven, 2001). Large individual differences exist in the amount of cognitive activity and in the content of thoughts by which people regulate their emotions in response to life events and trauma. Previous research distinguished between nine conceptually different cognitive emotion regulation strategies, i.e.: Self-blame, Other-blame, Rumination, Catastrophizing, Putting into Perspective, Positive Refocusing, Positive Reappraisal, Acceptance and Planning (e.g., Garnefski et al., 2001). Strong relationships have been found between these strategies and emotional outcomes, suggesting that strategies such as rumination, catastrophizing, and self-blame are maladaptive, and strategies such as positive refocusing and positive reappraisal are adaptive strategies (e.g., Garnefski & Kraaij, 2006, 2007; O’Driscoll, Laing, & Mason, 2014). In addition, there is evidence that specific cognitive strategies can moderate the relationship between life events and emotional problems. In a study on the relationship between bully victimization and emotional problems, it was shown that rumination and catastrophizing strengthened, while positive reappraisal and positive refocusing reduced the effects of bully victimization (Garnefski & Kraaij, 2014). Thus far, no studies are available that examined the role of cognitive emotion regulation strategies in the relationship between traumatic life events and somatic complaints.

The present study investigated relationships between the experience of traumatic life events, cognitive emotion regulation strategies, and somatic complaints. The first study question was: To what extent is the experience of a traumatic life event (such as loss and maltreatment) related to severity of somatic complaints? The experience of a traumatic life event was operationalized by a past negative life event that still produces strong and negative feelings in the present. If a relationship could be confirmed, it would suggest the importance of actively asking a patient with somatic complaints for the presence of unresolved traumatic memories. The second question was: To what extent do specific cognitive emotion regulation strategies explain variance of somatic complaints, over and above the experience of a traumatic life event. To determine whether associations would be attributable to the indicated traumatic life event and not to the accumulation of life events, we controlled for the total number of negative life events lifetime. It was expected that significant amounts of the variance in somatic complaints would be explained both by traumatic life events and cognitive emotion regulation strategies. More specifically, rumination, catastrophizing and self-blame were expected to be positively related, while positive reappraisal was expected to be negatively related to somatic complaints scores; In addition, we examined whether, over and above the direct effects, specific cognitive strategies moderated the relationship between traumatic life event and somatic complaints. We expected that high scores on rumination and catastrophizing would significantly strengthen, while high scores on positive reappraisal and positive refocusing would significantly reduce, the effect of the traumatic life event on somatic complaints. With regard to the other cognitive strategies (self-blame, other-blame, putting into perspective, acceptance and planning), we explored the possibility of moderation. Based on the literature, no specific hypotheses could be formulated for this relationship.

## Method

### Sample

The sample consisted of 465 18-to-65-year-old adults from a general population sample (mean age 45.5; *SD* = 13.0) recruited from a general practitioner’s (GP) practice in The Netherlands. Of the respondents, 81.5% was female, 50.0% was married or living together with a partner, 83.4% had received higher education.

### Procedure

Three GP practices in The Netherlands were approached to ask for permission to recruit patients who might be willing to participate in this research study. One GP practice agreed to participate. Although no formal data is available concerning the representativeness of this GP’s patient population compared with the general population in The Netherlands, the location of the practice is in a neighbourhood where the majority of people can be characterized by high education, high income, and a Dutch ethnic identity. All patients of the GP’s practice that met the following inclusion criteria were eligible for participation: Being between 18 and 65 years of age, being able to read and understand Dutch, and having shared an e-mail address with the GP. In total, 1850 persons met these criteria. Potential participants were contacted by an e-mail message that contained a link to an online self-report questionnaire. To preserve patients’ anonymity, i.e., to keep the researchers, the GP, and the practice staff blind with respect to the identity of both participants and non-participants, the e-mail was sent by the GP practice, and the questionnaire did not request the participant’s name. The e-mail message explained Leiden University was conducting a research project in cooperation with the GP practice, and that the project was focused on the relationships between the experience of negative life events, physical health, and psychological problems. It was explained that filling in the questionnaire would take about 25-min and that participation was voluntary. No compensation was offered for participation. Participants also filled out an informed consent as part of the questionnaire. Ethical approval had been obtained from the ethics committee of the University. A total of 465 persons completed the on-line questionnaires, which was 25.1% of the total number of e-mail messages that were sent to potential participants. There was no opportunity to gather data from those individuals who did not respond to the e-mail message.

### Instruments

#### Cognitive Emotion Regulation Strategies

To measure the cognitive strategies that participants used in response to negative life events, the Cognitive Emotion Regulation Questionnaire (CERQ) was used (Garnefski, Kraaij, & Spinhoven, 2001, 2002). The CERQ is a 36-item questionnaire, consisting of the following nine conceptually distinct subscales, each consisting of four items referring to what someone thinks after the experience of threatening or stressful life events:

1. *Self-blame*, referring to thoughts of blaming oneself for what one has experienced. Typical subscale items are: “I feel that I am the one to blame for it” and “I feel that I am the one who is responsible for what happened.”
2. *Acceptance*, referring to thoughts of accepting what one has experienced and resigning oneself to what has happened. Typical subscale items are: “I think that I have to accept that this has happened” and “I think that I cannot change anything about it.”
3. *Rumination or focus on thought*, referring to thinking about the feelings and thoughts associated with negative events. Typical subscale items are: “I often think about how I feel about what I have experienced” and “I dwell upon the feelings the situation has evoked in me.”
4. *Positive refocusing*, referring to thinking about joyful and pleasant issues instead of thinking about the actual events. Typical subscale items are: “I think of pleasant things that have nothing to do with it” and “I think of something nice instead of what has happened.”
5. *Refocus on planning*, referring to thinking about what steps to take and how to handle the negative events. It is the cognitive part of action-focused coping, which does not automatically imply that actual behavior will follow. Typical subscale items are: “I think about how to change the situation” and “I think about a plan of what I can do best.”
6. *Positive reappraisal*, referring to thoughts of attaching a positive meaning to the events in terms of personal growth. Typical subscale items are: “I think I can learn something from the situation” and “I look for the positive sides to the matter.”
7. *Putting into perspective*, referring to thoughts of playing down the seriousness of the events or emphasizing its relativity when compared to other events. Typical subscale items are: “I think that it all could have been much worse” and “I tell myself that there are worse things in life.”
8. *Catastrophizing*, referring to thoughts of explicitly emphasizing the terror of the experiences. Typical subscale items are: “I keep thinking about how terrible it was what I have experienced” and “I continually think how horrible the situation has been.”
9. *Blaming others*, referring to thoughts of putting the blame of what one has experienced on others. Typical subscale items are: “I feel that others are to blame for it” and “I feel that others are responsible for what has happened.”

The following instruction was provided: “Everyone gets confronted with negative or unpleasant events now and then and everyone responds to them in his or her own way. By the following questions you are asked to indicate what you generally think, when you experience negative or unpleasant events.” Response scales of the items ranged from: 1 = (*almost*) *never* to 5 = (*almost*) *always*. Individual subscale scores were obtained by summing up the scores belonging to the particular subscale (possible range per subscale: 4–20). Higher scores refer to higher use of the specific strategy.

Research on cognitive emotion regulation strategies, as measured by the CERQ, has shown that the subscales have good internal consistencies, with alphas ranging from 0.67 to 0.81 (Garnefski et al., 2001, 2002). In the present study alpha reliabilities of the subscales were the following: 0.83 for Self-blame, 0.76 for Acceptance, 0.83 for Rumination, 0.86 for Positive refocusing, 0.84 for Planning, 0.84 for Positive reappraisal, 0.83 for Putting into Perspective, 0.63 for catastrophizing, and 0.84 for Other-blame.

#### Somatic Complaints

To measure somatic complaints, one of the subscales of the SCL-90 was used, i.e., the Somatization subscale (Symptom Check List: Derogatis & Cleary, 1977; Dutch translation and adaptation by; Arrindell & Ettema, 1986). The following 12 somatic complaints were assessed: painful muscles, painful joints, painful limbs, difficulty breathing, abdominal complaints, stomach complaints, body weakness, fatigue complaints, heart complaints, and chest complaints. The following instruction was provided: “Below is a list of complaints that people sometimes have. Please read each one carefully. After you have done so, please indicate to what extent that specific complaint has bothered or distressed you during the past week, including today.”

Response scales of the items ranged from: 1 = *not at all* to 5 = *very much*. An individual scale score was obtained by summing up the responses (possible range: 4–20). Higher scores refer to a higher amount of somatic complaints.

Previous studies of the SCL-90 Somatization scale have reported alpha-coefficients ranging from 0.74 to 0.89. In addition, test–retest reliabilities are found to be good and both subscales have been found to show strong convergent validity with other conceptually related scales (Arrindell & Ettema, 1986). In the present study, a Cronbach’s alpha of 0.82 was found.

#### Traumatic Life Event with Current Impact

To assess whether participants have experienced a traumatic life event, they were asked to indicate whether there is a past negative life event that still produces strong and negative feelings in the present. The following instruction was provided: “Did you experience one or more negative life events in your life that still produce strong and negative feelings in the present?” Possible answers were: *yes* and *no*. If the answer was *yes*, participants were asked to describe the life event with the strongest negative feelings in the present. In the present study, this variable was included as a dichotomous variable (yes/no traumatic event).

#### Total Number of Lifetime Negative Life Events

The total number of lifetime negative life events was assessed. A checklist was used to collect data on the experience of negative life events (provided at http://www.cerq.leidenuniv.nl). The instruction was: “Did you experience one of the following events in your life, before the age of 16, between 16 and 1 year ago, and/or in the past year? If you did not experience a particular event in any of the three periods, please check the box *no*. If you experienced a particular event in a particular period, please check the box concerning the period in which the event occurred. If an event occurred in several periods, please check the event for all these periods.” Life events that were assessed were: sexual abuse (self), physical abuse (self), victim of crime (self), victim of bullying (self), violence within home situation, alcohol or drug abuse within home situation, suicide attempt by close relative, divorce (parents/self), death of close relative. If the *no* was checked (did not experience this life event in any of the periods), the response was scored as 0. If the *yes* box was checked for a specific period, the response was scored as 1. An individual total number of negative events score was obtained by summing up the responses (possible range: 0–27). Higher scores refer to a higher amount of negative life events.

### Statistical Analysis

Means, standard deviations and ranges of the study variables (cognitive emotion regulation strategies, somatic complaint score, total number of negative life events) were calculated. In addition, the number of participants that reported a traumatic life event with current impact was determined. Subsequently, associations between somatic complaints scores and background variables (gender, age, and number of life events) were calculated. The relationships between somatic complaints scores and gender were tested by *t* tests, the relationships between somatic complaints and age were tested by a Pearson correlation. Likewise, a Pearson correlation was calculated between somatic complaints and total number of negative life events. On basis of these analyses, it was decided that gender, age, and number of life events were to be included in the main multiple regression analysis.

Next, Pearson correlations between the experience of a traumatic life event, cognitive strategies, and somatic complaints were calculated. Finally, hierarchical multiple regression was used to answer questions about whether specific cognitive strategies explain variation in somatic complaints over and above the effect of the number of negative life events, and whether the relationship between traumatic life events and somatic complaints is moderated by specific cognitive strategies. This method makes it possible to determine the extent to which the specific blocks of predictor variables make a unique contribution to the prediction of somatic complaints after the contribution of preceding blocks of variables has been taken into account. Somatic complaints was the dependent variable.

As gender, age, and number of number of negative life events proved to be significantly related to somatic complaints, these variables were entered respectively in the first (gender and age) and second step (life events). In the third step, *traumatic life event* was entered. In the fourth step the nine cognitive strategies were entered. In the fifth step, the nine interactions between traumatic life event and cognitive emotion regulation strategies were entered. Because the interaction terms were included, all variables were centered before being entered into the regression equation.

## Results

Table 1 presents descriptive statistics for the study variables. In total, 232 respondents (49.9%) reported a traumatic life event that still produces strong and negative feelings in the present. With regard to the 232 reported traumatic life events with current impact, it can be added that: 110 events concerned the loss or threat of loss (e.g., death, suicide, serious disease of close relative, divorce); 60 of the reported events referred to maltreatment (sexual abuse, physical abuse, emotional maltreatment, bullying); and 23 of the other reported events mostly referred to (unexpected) events in a relationship, at home, or at work (e.g., suddenly being fired, cheating by partner, house on fire, burglary). In 34 cases the participant indicated that there was a traumatic event, but that they did not wish to disclose the details.

**Table 1:** Descriptive statistics for total number of life events, somatic complaints, and cognitive emotion regulation scales (*N* = 465)

Variables	*M* (*SD*)	Actual range	Possible range	*N* (%)
Total number of life events	2.55 (2.21)	0–11	0–27	
Traumatic event with current impact	–	–	–	**yes**: 232 (49.9%)
Somatic complaints total score	18.86 (6.19)	12–48	12–60	
Cognitive emotion regulation strategies				
Selfblame	8.87 (3.55)	4–20	4–20	
Other blame	5.72 (2.13)	4–20	4–20	
Acceptance	11.75 (3.41)	4–20	4–20	
Rumination	10.40 (3.72)	4–20	4–20	
Catastrophizing	5.48 (1.79)	4–17	4–20	
Planning	13.58 (3.60)	4–20	4–20	
Putting into perspective	11.51 (3.81)	4–20	4–20	
Positive reappraisal	12.58 (3.86)	4–20	4–20	
Positive refocusing	11.14 (3.69)	4–20	4–20	

The relationships between background variables gender and age with somatic complaints scores were evaluated with *t* tests. Women (*M* = 19.20, *SD* = 6.23) reported significantly more somatic complaints than men (*M* = 17.37, *SD* = 5.84), *t*(461) = 2.48, *p* < .05. In addition, a significant Pearson correlation was found between age and somatic complaints (*r* = .13, *p* < .01). Additionally, the Pearson correlation between total number of negative life events and somatic complaints was 0.32 (*p* < .05). Therefore, gender, age, and total number of life events were included as control variables in the subsequent multiple regression analysis (MRA).

Table 2 presents the bivariate Pearson correlations between all study variables. It was shown that somatic complaints had a Pearson correlation of 0.36 with the reporting of a traumatic life event. Number of life events and traumatic event had a correlation of 0.24. In addition, significant Pearson correlations were found between somatic complaints and various cognitive emotion regulation strategies; the strongest relationships were observed with self-blame, rumination, and catastrophizing. Further, low to moderate correlations were observed among the cognitive emotion regulation strategies.

**Table 2:** Pearson correlation coefficients for study variables

Variables	Som	Nle	Tle	1	2	3	4	5	6	7	8	9
Somatic complaints (Som)	–											
Total number of life events (Nle)	0.32***	–										
Traumatic life event (Tle)	0.36***	0.24***	–									
Cognitive emotion regulation strategies												
1. Selfblame	0.29***	0.07	0.17***	–								
2. Otherblame	0.16***	0.16***	0.18***	0.16**	–							
3. Acceptance	0.21***	0.11*	0.18***	0.24***	0.11*	–						
4. Rumination	0.31***	0.10*	0.27***	0.39***	0.21***	0.32***	–					
5. Catastrophizing	0.29***	0.16***	0.39***	0.18***	0.32***	0.17***	0.37***	–				
6. Planning	0.07	0.03	−0.01	0.25***	0.19***	0.42***	0.45***	0.05	–			
7. Putting into perspective	−0.04	−0.09*	−0.18***	0.14**	− 0.06	0.31***	−0.01	−0.18***	0.33***	–		
8. Positive reappraisal	−0.07	0.06	−0.20***	0.04	−0.10*	0.32***	0.20***	−0.16***	0.53***	,46***	–	
9. Positive refocusing	−0.08	0.00	−0.04	−0.18***	0.03	0.21***	−0.17***	−0.15**	0.25***	0.32***	0.39***	–

***p < .001, ** p < .01, *p < .05

Table 3 presents results of a hierarchical MRA with total somatic complaints score as the dependent outcome variable. Within Table 3, the order of steps reflects the sequence in which individual predictor variables and groupings of predictor variables, were included in the prediction model. The beta coefficients at each step indicate whether a specific predictor included in the model at a specific step accounted for a significant increase in the amount of variation in somatic complaints scores. In the first step, the background variables, gender and age, were entered as predictors, and together accounted for a significant amount of variance, *R* ^2^ = 0.02; *F*(2,452) = 4.40, *p* < .05, although gender appeared to be the primary source of that result. In the second step, total number of life events was entered into the regression analysis; the addition of that predictor resulted in a significant increase in the amount of variation in somatic complaints that was accounted for, *R* ^2 ^_change_ = 0.10; *F*(1,453) = 29.37, *p* < .001. In the third step, traumatic life event was entered as a predictor; it added significantly to the amount of variance in somatic complaints that was explained, *R* ^2 ^_change_ = 0.08; *F*(1,452) = 45.94, *p* < .001. In the fourth step, the nine cognitive emotion regulation strategies were entered; as a block, these predictors explained a significant amount of additional variance in somatic complaints, *R* ^2 ^_change_ = 0.09, *F*(9.443) = 6.40, *p* < .001, although it was the strategies of selfblame, rumination, and catastrophizing that seemed primarily responsible for the increase in variance accounted for. In the fifth step, nine interactions were tested to determine whether the associations between cognitive emotion regulation strategies and somatic complaints were different depending on the presence or absence of a *traumatic life event*, i.e., one that has current emotional impact. However, this additional step was non-significant, *R* ^2 ^_change_ = 0.01, *F*(9,434) = 0.89, *p* = .54. These interaction terms were therefore omitted from the final model.

**Table 3:** Hierarchical MRA on somatic complaints score

Steps/blocks and variables	Step 1	Step 2	Step 3	Step 4
	ß	ß	ß	ß
First step/block				
Gender	0.10*	0.10*	0.09*	0.10*
Age	0.02	0.02	0.03	0.06
Second step/block				
Number of life events		0.32***	0.24***	0.22***
Third step/block				
Traumatic life event			0.30***	0.20**
Fourth step/block				
Cognitive emotion regulation strategies				
1. Selfblame				0.18***
2. Otherblame				0.01
3. Acceptance				0.09
4. Rumination				0.11*
5. Catastrophizing				0.11*
6. Planning				−0.07
7. Putting into Perspective				0.04
8. Positive Reappraisal				−0.09
9. Positive Refocusing				0.01

***p < .001, **p < .01, *p < .05

The total amount of variance explained in the final model (after the fourth step) was 28.9%, *F*(13,443) = 13.88, *p* < .001. Throughout all steps, gender retained a small significant, positive effect, indicating that women have more somatic complaints than men. Additionally, both the number of life events and the reporting of a traumatic event added significantly to the variance that was explained. Over and above these effects, the cognitive strategies of self-blame, rumination, and catastrophizing significantly added to the model, i.e., the more these strategies were used, the more somatic complaints were reported.

## Discussion

Our study confirms that somatic complaints were significantly related to the reporting of a past traumatic life event that has current emotional impact (such as loss and maltreatment), even beyond the total number of negative life events experienced in a lifetime. The study also confirms that somatic complaints are significantly related to maladaptive cognitive coping strategies, especially: blaming oneself, ruminating, and catastrophizing about negative life events. No evidence was found, however, for the hypothesis that specific cognitive strategies would moderate the relationship between somatic complaints and a traumatic life event with current impact.

With regard to the relationship between somatic complaints and traumatic life events, other studies have indicated that traumata may play an important role in the development and maintenance of somatic complaints (Brown, 2004, 2006; Roelofs & Spinhoven, 2007; Afari et al., 2014). Results of the present study add that this relationship is confirmed even by asking only one simple question about the presence of unresolved traumatic memories. This suggests the value of actively asking all patients with somatic complaints about the presence of unresolved traumatic memories (like loss or maltreatment; one could provide examples). The question provided in the present study is a very practical, short and to-the-point question, that can be asked by any treatment provider. If someone actually reports such a traumatic life event with current impact, a second step could be to refer the patient to a psychologist or psychiatrist for further assessment, and if indicated, trauma treatment.

In addition, we showed that, next to the traumatic life experience itself, certain maladaptive cognitive coping strategies, such as self-blaming, ruminating, and catastrophizing were related to present somatic complaints. Previous studies have demonstrated that persons who engage in extensive self-blaming, ruminating, and catastrophizing about negative events are likely to be more vulnerable to emotional problems such as depression and anxiety than others. The present results extend this line of reasoning by indicating that persons who make extensive use of these same three cognitive strategies are also more vulnerable to presenting with somatic complaints than persons who use these strategies less often. Assessing people’s coping styles, and especially the tendency to blame oneself, to ruminate and/or to catastrophize might therefore also suggest targets for intervention. People could be assisted to challenge these maladaptive cognitive coping patterns and to acquire new, more adaptive strategies. Although specific interventions on changing cognitive coping strategies are not available yet, these could easily be integrated into existing coping skills trainings and/or cognitive behavioral therapies, which are widely used and evidence based (Hofmann, Asnaani, Vonk, Sawyer, & Fang, 2012).

It is important to mention some limitations of the study. One issue was that the detection of somatic complaints was made by the SCL-90 Somatization subscale, which can be considered as a general measure of symptoms of physical distress, but which does not make it possible to distinguish complaints that have clear medical causes from those that do not. We also do not have additional data that could provide information about the cause or background of the somatic complaints, e.g., whether or not a medical/organic cause has been identified. Future studies should include more specific instruments or data that would be able to distinguish between somatic complaints with and somatic complaints without a medical or organic cause. Another issue for the present study was our operationalization of a traumatic life event by use of a single question that asked participants to identify a “past negative life event that still produces strong and negative feelings in the present.” Although this operationalization is appealing because of its simplicity, brevity, and ease of use in any GP or other (medical) practice, answers to the question may be subject to over- or underreporting due to subjective differences in interpretation or reference standards. With this measure, no objective conclusions can be drawn about the experience of traumata or the presence of PTSD, which would require more intensive and professional clinical assessment. Generally speaking, to be able to draw firm conclusions about the contribution of traumatic life events and cognitive coping strategies contributing to risk of somatic complaints, more qualitative methods could be applied; for example, a clinician might use in-depth interviews, narrative measures, or daily diaries.

Another important limitation is associated with our study sample, which comes from only one GP practice. Although official data were not available with regard to the representativeness of this GP’s patient population, it was assumed (because of the location of the practice) that people with a higher economic status were overrepresented. This would limit the generalizability of the results to persons from lower economic classes and other cultural or ethnic backgrounds. Although all patients of the GP practice were invited, regardless of medical status and conditions, the response rate was low and data from non-responders was not available. Therefore, we also do not know the extent to which the sample was representative for the GP practice, itself. There might be a response bias; for example, people with a more serious or chronic medical condition might be overrepresented in the sample. The possible role of such factors cannot be determined because no information could be obtained from persons who did not participate. In addition, most participants were female and had higher education, which limits generalization to men and to those who are less educated. Another issue is that the results of the present study are based on cross-sectional data. Therefore, it is important to acknowledge that no conclusions can be drawn about causality or directions of influence. For purposes of generalizability, the study should be repeated with other samples and with other methods, while prospective elements should be included. In addition, it is important to acknowledge that trauma and cognitive factors are not the only factors that are important in relation to somatic complaints. It would be a worthy challenge to set up future research studies that would study a wider range of factors, e.g., as psychological, medical/biological, genetic, and social factors in a single investigation.

Notwithstanding the above limitations, our results suggest the potential value of clinicians inquiring about unresolved traumatic memories and methods for coping with such memories as a way to improve management of patients with somatic complaints that have no clear medical explanation. Further studies using other samples and other research methods would be useful for assessing the clinical value of this approach to working with these patients.
